# The Association of BAG6 with SGTA and Tail-Anchored Proteins

**DOI:** 10.1371/journal.pone.0059590

**Published:** 2013-03-22

**Authors:** Pawel Leznicki, Quentin P. Roebuck, Lydia Wunderley, Anne Clancy, Ewelina M. Krysztofinska, Rivka L. Isaacson, Jim Warwicker, Blanche Schwappach, Stephen High

**Affiliations:** 1 Faculty of Life Sciences, University of Manchester, Manchester, United Kingdom; 2 Department of Biochemistry I, University of Göttingen, Göttingen, Germany; 3 Division of Molecular Biosciences, Imperial College London, London, United Kingdom; Institute of Molecular and Cell Biology, Singapore

## Abstract

**Background:**

The BAG6 protein is a subunit of a heterotrimeric complex that binds a range of membrane and secretory protein precursors localized to the cytosol, enforcing quality control and influencing their subsequent fate.

**Methodology and Principal Findings:**

BAG6 has an N-terminal ubiquitin-like domain, and a C-terminal Bcl-2-associated athanogene domain, separated by a large central proline-rich region. We have used *in vitro* binding approaches to identify regions of BAG6 important for its interactions with: i) the small-glutamine rich tetratricopeptide repeat-containing protein alpha (SGTA) and ii) two model tail-anchored membrane proteins as a paradigm for its hydrophobic substrates. We show that the BAG6-UBL is essential for binding to SGTA, and find that the UBL of a second subunit of the BAG6-complex, ubiquitin-like protein 4A (UBL4A), competes for SGTA binding. Our data show that this binding is selective, and suggest that SGTA can bind either BAG6, or UBL4A, but not both at the same time. We adapted our *in vitro* binding assay to study the association of BAG6 with an immobilized tail-anchored protein, Sec61β, and find both the UBL and BAG domains are dispensable for binding this substrate. This conclusion was further supported using a heterologous subcellular localization assay in yeast, where the BAG6-dependent nuclear relocalization of a second tail-anchored protein, GFP-Sed5, also required neither the UBL, nor the BAG domain of BAG6.

**Significance:**

On the basis of these findings, we propose a working model where the large central region of the BAG6 protein provides a binding site for a diverse group of substrates, many of which expose a hydrophobic stretch of polypeptide. This arrangement would enable the BAG6 complex to bring together its substrates with potential effectors including those recruited via its N-terminal UBL. Such effectors may include SGTA, and the resulting assemblies influence the subsequent fate of the hydrophobic BAG6 substrates.

## Introduction

Tail-anchored (TA) proteins have provided a convenient paradigm for studying post-translational membrane insertion at the endoplasmic reticulum [1]. Whilst there are multiple pathways for the delivery of TA proteins to the endoplasmic reticulum [2], dictated in part by the hydrophobicity of the tail-anchor domain [3], [4], most recent studies have focused on a pathway that is dependent upon the 40 kDa component of the transmembrane domain recognition complex (TRC40) [1], [5], [6]. Mammalian TRC40 was first identified as a TA protein interacting partner using *in vitro* assays [7], [8], and its binding to a precursor reflects commitment to ER delivery via an interaction that relies on a membrane protein receptor composed of the tryptophan-rich basic protein (WRB) [9], and the calcium-modulating cyclophilin ligand (CAML) [10]. This part of the TA protein delivery pathway to the ER is highly conserved, and in *Saccharomyces cerevisiae* a similar process is mediated by the TRC40 homolog Get3, and the heteromeric Get1/Get2 membrane receptor [1], [6], [11],[12],[13]. In higher eukaryotes, the binding of TA protein substrates to TRC40 relies on their prior association with an upstream loading factor, the BAG6-complex, which is comprised of BAG6 (Bat3, Scythe), TRC35 (35 kDa component of the transmembrane domain recognition complex, also known as mammalian Get4, C7orf20 and conserved edge-expressed protein) and UBL4A (Ubiquitin-like protein 4A or mammalian Get5) [14], [15]. Likewise, in yeast the binding of TA proteins to Get3 also involves a prior association with an upstream loading complex that in this case is comprised of Get4, Get5 and Sgt2 [16], [17], [18]. Interestingly SGTA, the mammalian ortholog of Sgt2, is a well known interacting partner of BAG6 [19], [20], and a targeted proteomic analysis placed both BAG6 and SGTA into an interaction network that also included UBL4A, TRC35 and TRC40 [21]. Three recent studies have confirmed a direct physical interaction between SGTA/Sgt2 and UBL4A/Get5 [22], [23], [24], and SGTA can also bind to the hydrophobic region of TA proteins *in vitro* [14], [25].

Shortly after its role in TA protein delivery via the TRC40 pathway was identified, several studies indicated a more extensive role for BAG6 consistent with earlier genetic evidence of a link between components of the yeast GET pathway and the ubiquitin proteasome system [26]. Firstly, BAG6 was shown to be required for the ubiquitin dependent degradation of aberrant nascent chains upon their release from the ribosome [27]. Secondly, the BAG6-complex was found to facilitate the ER associated degradation (ERAD) of certain aberrant membrane proteins [28], [29], a process that SGTA also contributes to in concert with BAG6 [24]. Lastly, BAG6 was shown to play an important role in the ubiquitination and degradation of mislocalized membrane and secretory proteins that fail to reach the ER and remain in the cytosol [30]. Strikingly, this latter process can be antagonized by SGTA which acts to promote the deubiquitination of mislocalized membrane proteins, and hence promote their stability [31]. On the basis of these studies, a viable working hypothesis is that the BAG6-complex can recognize a range of “hydrophobic” substrates located in the cytosol and provide a sorting step that ensures they are correctly assigned to appropriate down stream effectors. These effectors may either enable the subsequent delivery of substrates to the ER for membrane insertion (via TRC40) or facilitate their ubiquitination and degradation at the proteasome (via components of the ubiquitin proteasome system) [30], [32]. In this study we have investigated the importance of the N-terminal UBL domain (∼ residues 17 to 88) and C-terminal BAG domain (∼ residues 1049 to 1105) of the BAG6 protein for binding to SGTA and two tail-anchored protein substrates. The BAG6-UBL is essential for binding to SGTA, and the UBLs from the UBL4A subunit of the BAG6-complex, and even its *S. cerevisiae* homolog, Get5, can also bind to SGTA. In contrast, two other UBL-containing proteins and ubiquitin show no such interaction, and the C-terminal BAG domain has no role in SGTA binding. Competition experiments suggest that SGTA can associate with either one copy of BAG6, or one copy of UBL4A, but not both at the same time. The UBL and BAG domains are dispensable for the *in vitro* binding of BAG6 to the hydrophobic transmembrane domain of the model TA protein, Sec61β. Likewise, both domains are also irrelevant for the BAG6 dependent nuclear relocalization of a second model TA protein, GFP-Sed5, in yeast. On the basis of this study, we propose that the BAG6-complex provides a link between hydrophobic substrates and a range of potential effectors, and suggest a working model to describe this role.

## Results

### The BAG6 UBL Binds SGTA

**Figure 1:**
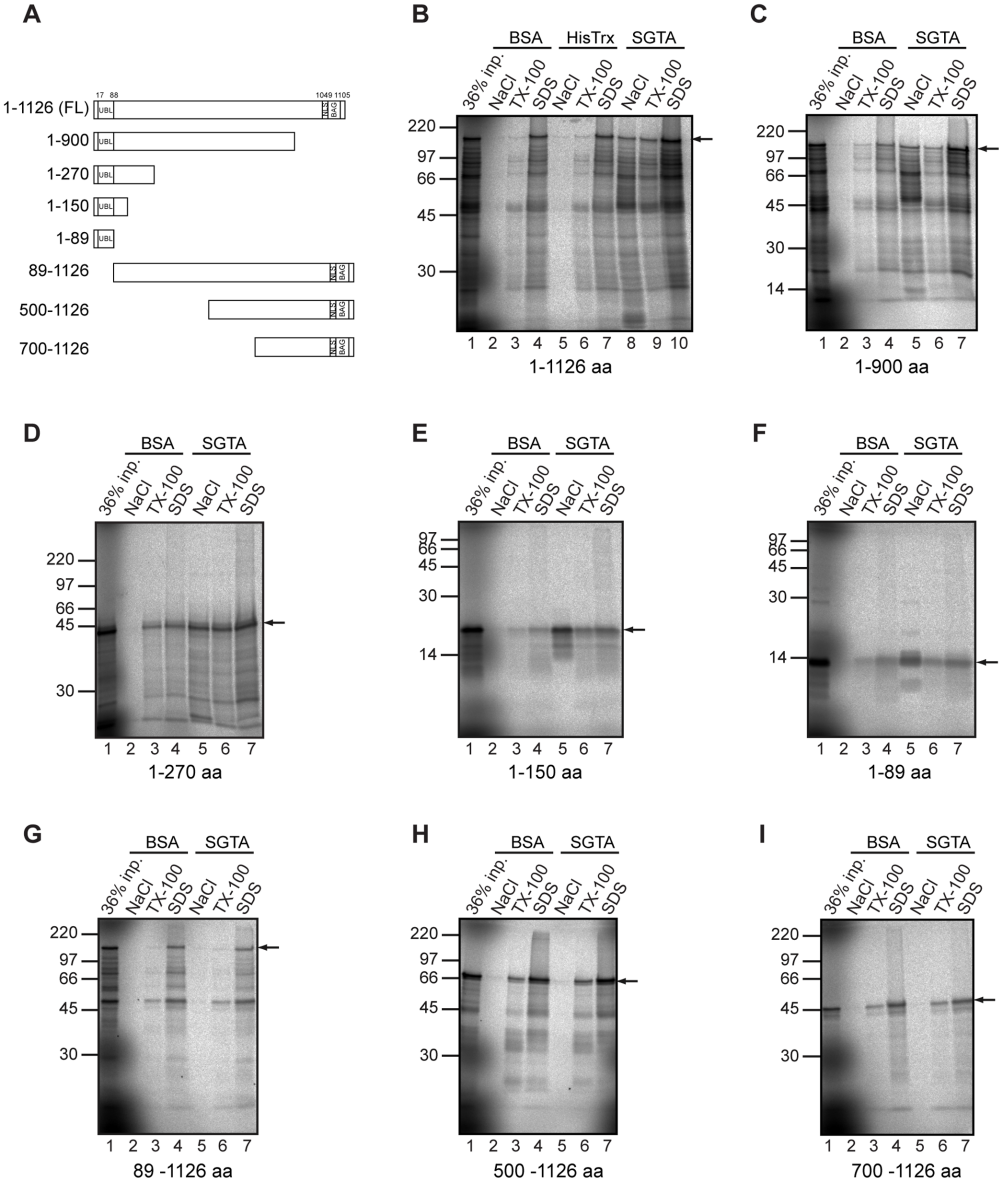
BAG6 deletion mutants display differences in SGTA binding. As with our previous analysis [14], we employed isoform 2 of BAG6, which lacks residues 185–190 of the canonical isoform 1 (see Uniprot P46379) in this work. An outline of full-length (FL) BAG6 (isoform 2) and truncations used to study SGTA binding is shown (A). UBL indicates the ubiquitin like domain, NLS a nuclear localization signal and BAG a Bcl-2-associated athanogene domain. Radiolabelled forms of full-length BAG6 (B), or a range of fragments as indicated (C to I), were synthesized *in vitro* using a wheat-germ extract and incubated with immobilized BSA, His-thioredoxin (His-Trx) or SGTA as shown (see Materials and Methods). Beads were isolated and washed before sequentially eluting bound material with high-salt (NaCl), Triton-X100 (TX-100) and finally SDS-PAGE (SDS) sample buffer as indicated. Eluted material was resolved by SDS-PAGE, together with a sample of the input (equivalent to 36% of the amount added to the pull down assay), and products were visualized by phosphorimaging. An arrow indicates the location of the relevant translation product in each of the reactions. The abnormal migration of the fragment comprising residues 1–270 of BAG6 (see panel D) most likely reflects the comparatively high proportion of proline residues in the first half of the protein (see also Figure S3).

In order to investigate the interaction of BAG6 with SGTA, we established an *in vitro* binding assay, utilizing immobilized SGTA as the bait and BAG6 generated by cell free translation as its potential interacting partner. Since both SGTA and homologous components of the BAG6 complex are present in reticulocyte lysate [14], [15], we employed a wheat germ translation system in an attempt to minimize any potential contribution from endogenous components to the *in vitro* interactions being analyzed. In order to detect binding, we used previous comparable studies [8], [14] as a starting point to develop a protocol that involved extensive washing of the beads to which the relevant bait, or a suitable control, had been coupled. Specific interactions were then disrupted using sequential elution with high-salt and then Triton-X100, and then finally any remaining material was recovered from the beads using SDS-PAGE sample buffer (see Figure S1). When full length, radiolabelled BAG6 (isoform 2, see Figure 1A) was synthesized, the full-length product showed specific salt sensitive binding to immobilized SGTA, but not to immobilized BSA or immobilized His-Trx, that were both employed as controls (Figure 1B, cf. lanes 2, 5 and 8, see arrow). This behavior of full length BAG6 was highly reproducible (also see Figures S2A and S2B), and a range of shorter, truncated, BAG6 products that result from the *in vitro* translation system behaved in a similar fashion (Figure 1B, cf. lanes 2, 5 and 8; Figures S2A and S2B). We employed this salt sensitive binding as the basis for a deletion mapping study to identify any defined region(s) of BAG6 that are important for SGTA binding. Progressive deletions from the C-terminus of BAG6 did not perturb its salt sensitive binding to immobilized SGTA at a qualitative level (Figures 1C to 1E, cf. lanes 2 and 5), and even the N-terminal 89 residues alone showed clear binding in this assay (Figure 1F, lanes 2 and 5). Previous studies had implicated an N-terminal region of BAG6 in its association with SGTA [19], [20], and we found that the deletion of the N-terminal 88 residues alone, including a predicted UBL encompassing residues 17 to 88 (Figure 1A), resulted in a complete loss of salt sensitive binding (Figure 1G, cf. lanes 2 and 5). Likewise the removal of larger portions of the N-terminal region of BAG6 also prevented the specific interaction of the resulting truncated polypeptide with immobilized SGTA (Figures 1H and 1I, cf. lanes 2 and 5). The behavior of the BAG6 deletion constructs in this in vitro binding assay was also highly reproducible (see Figures S2C to S2G), and we studied several additional truncated forms of BAG6 which also behaved in an entirely consistent manner (Figures S3A to S3D), On the basis of this analysis, we conclude that the N-terminal region of BAG6, and specifically the N-terminal UBL domain is required for its efficient binding to SGTA (cf. Figure 1A).

**Figure 2:**
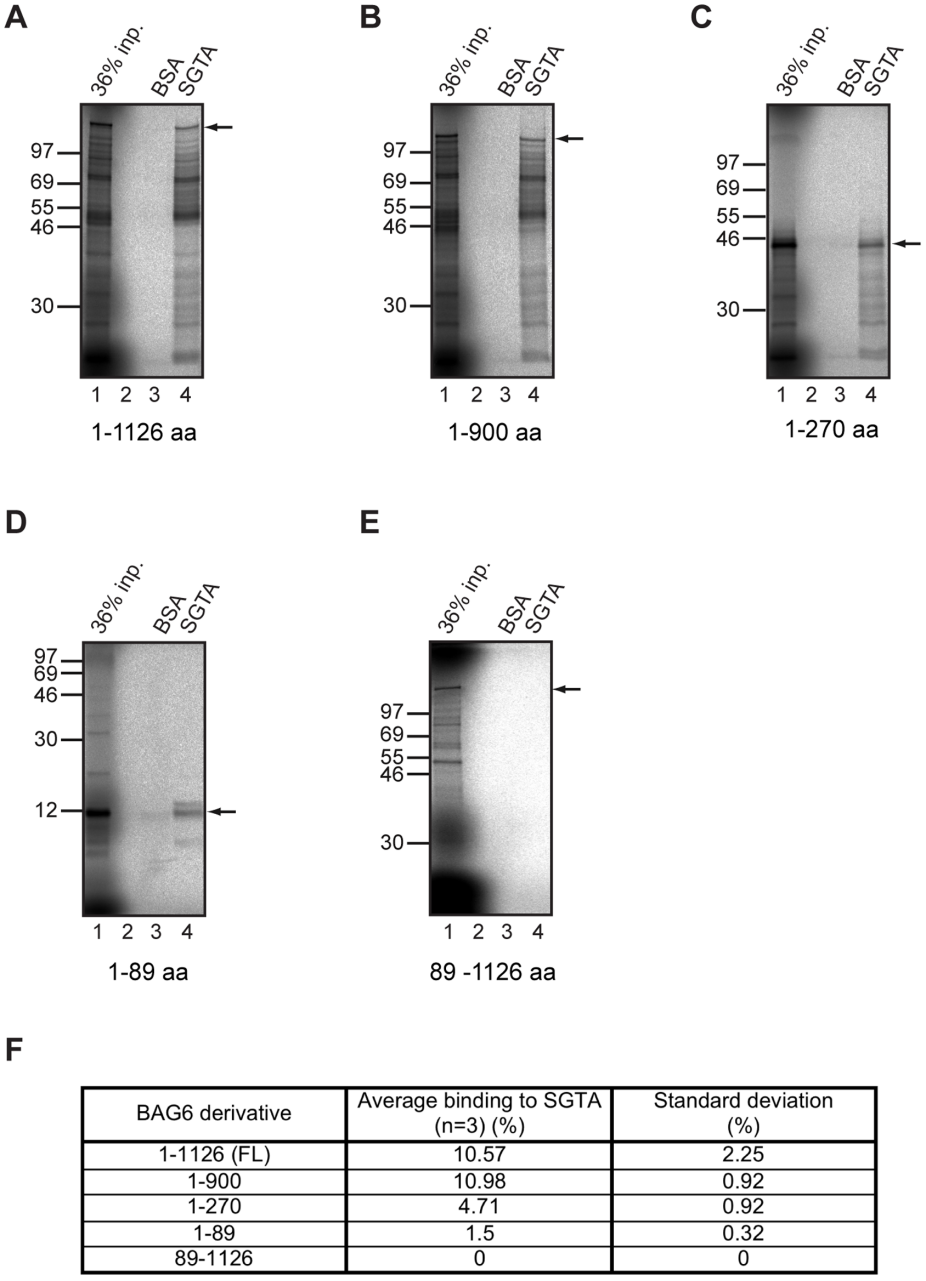
The N-terminal region of BAG6 is required for SGTA binding. The salt sensitive binding of full-length BAG6 (A) and selected deletion mutants (B to E) to SGTA was quantified by subtracting the signal recovered with immobilized BSA (non-specific) from that recovered with immobilized SGTA (specific), and expressing the SGTA bound fraction as a percentage of the signal obtained for the input. In each case it was the major translation product that was quantified by phosphorimaging (see arrow). The values presented for each BAG6 derivative (F) are derived from three independent experiments (n = 3) and indicate the standard deviation.

We took advantage of the fact that the BAG6 polypeptides used for the SGTA pull down assay are radiolabelled, and carried out a series of pull down experiments where we quantified the proportion of material bound in a salt sensitive manner. This quantitative approach revealed that whilst all fragments with an intact N-terminal UBL bind to SGTA, the relative efficiency of their recovery is variable (Figure 2). Hence, full length BAG6 and the N-terminal 900 residues, showed comparable levels of binding at ∼11% (Figure 2F). Binding efficiency was reduced to ∼5% for the N-terminal 270 residues whilst the first 89 residues showed ∼1.5% recovery of the input (Figure 2F). As before, in the absence of the N-terminal UBL, no binding was detectable (Figure 2F, see 89–1126). Taken together these data suggest that the C-terminal BAG domain is completely dispensable for BAG6 binding to SGTA (cf. Figure 1A), whilst the N-terminal UBL domain is essential for this interaction. The enhanced recovery of the longer BAG6 fragments (>270 residues) may reflect improved folding of the UBL and/or additional contributions by other elements within these polypeptides (see Discussion).

### SGTA Binds Selected UBLs

**Figure 3:**
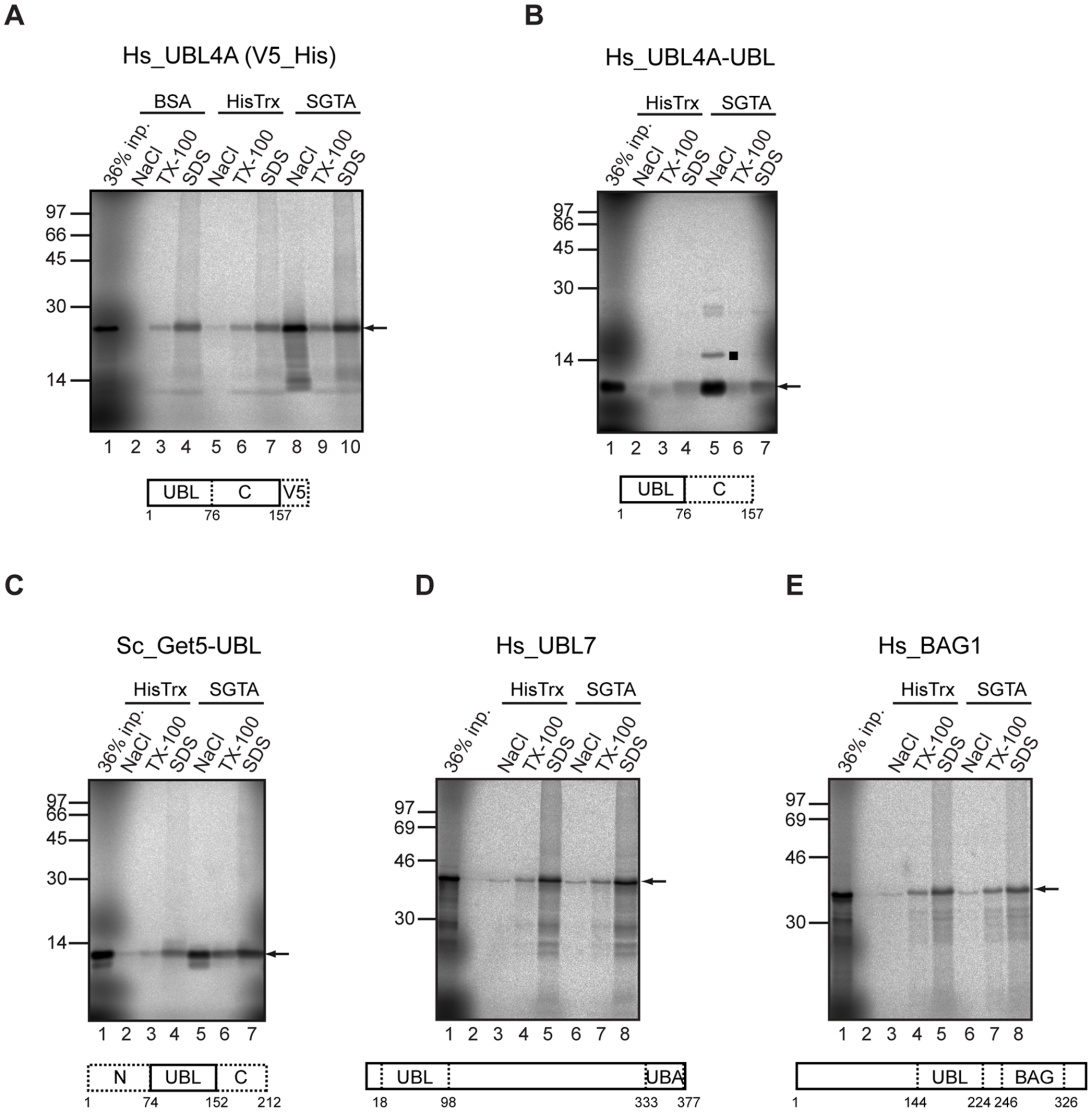
SGTA only binds selected ubiquitin like domains. The binding of radiolabelled versions of full-length human UBL4A with a C-terminal V5 tag (A), its UBL domain alone (B), the UBL domain of *S. cerevisiae* Get5/Mdy2 (C), full-length human UBL7 (D) and full-length human BAG1 (E) was analyzed as described for Figure 1. The filled square (panel B) indicates a minor radiolabelled species derived from the UBL4A-UBL domain that appears to be enriched in the SGTA associated fraction. It may represent a posttranslational modification of the UBL4A fragment, although its precise origin remains to be determined. An arrow indicates the location of the relevant translation product in each of the reactions.

In *S. cerevisiae*, Sgt2, an ortholog of SGTA, is known to bind Get5 via its N-terminal UBL [17], [33], [34], a situation that resembles the SGTA-BAG6 interaction detailed above. UBL4A is the presumptive mammalian homolog of Get5 [1], [18], and we speculated that it might also bind SGTA via its UBL domain. We therefore employed the same SGTA-dependent pull-down assay to address this issue, and found a striking interaction between *in vitro* synthesized UBL4A bearing a C-terminal V5/poly-histidine tag and immobilized SGTA that was both specific and salt sensitive (Figure 3A, cf. lanes 2, 5 and 8, see arrow). This interaction could be recapitulated with the N-terminal UBL region of UBL4A alone (Figure 3B, lanes 2 and 5, see arrow). Given the homology between mammalian and yeast components involved in TA protein biogenesis [1], [35], we went on to analyze the interaction of the UBL domain from *S. cerevisiae* Get5 with mammalian SGTA. Once again clear evidence of binding was observed (Figure 3C, lanes 2 and 5; see also Figure S3E). UBLs are found in a range of different proteins, and we next asked whether SGTA was simply a ubiquitous UBL binding factor by analyzing two more UBL containing proteins that have no known link to TA protein targeting. We chose to investigate UBL7 since its UBL is close to the N-terminus of the protein, like that of BAG6 and UBL4A, whilst BAG1 resembles BAG6 by containing both a UBL and a BAG domain. In contrast to the robust interactions of SGTA with BAG6, UBL4A and the Get5-UBL, we found no indication of any specific interaction with either UBL7 (Figure 3D, cf. lanes 3 and 6) or BAG1 (Figure 3E, cf. lanes 3 and 6).

**Figure 4:**
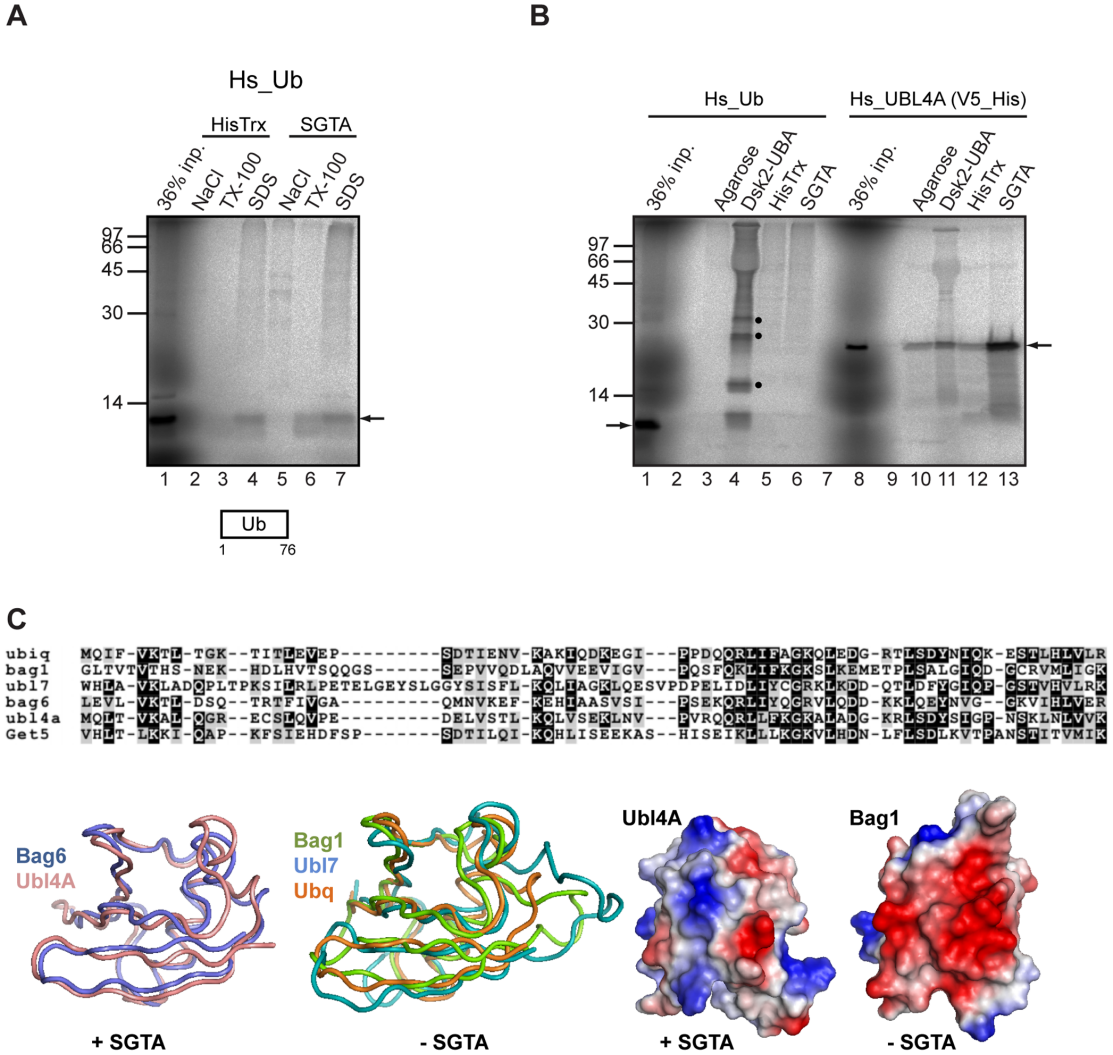
SGTA does not bind ubiquitin. The ability of *in vitro* synthesized human ubiquitin to bind immobilized SGTA (A) was analyzed as for Figure 1. Radiolabelled ubiquitin was also analyzed for binding to the Dsk2-UBA domain under altered binding conditions that favor this interaction (B), see Materials and Methods. Ubiquitin binding to immobilized SGTA and two control resins, together with a parallel experiment looking at tagged UBL4A binding, were also performed under the same conditions (B). Filled circles indicate potential oligomeric forms of ubiquitin that form during synthesis, whilst other symbols are as previously defined. In order to look for possible structural determinants of UBLs that are competent for SGTA binding (C), a structural alignment of ubiquitin (PDB id 3ons), BAG1 (1wxv), UBL7 (1×1 m), BAG6 (1 w×9), and UBL4A (2 dzi) was made, and the Get5 UBL aligned to these other proteins using Clustal (panel C, top section). The sequence alignment indicates identical and conserved amino acid residues that are present at specific locations (black and gray squares respectively). The only substantial deviations from the base ubiquitin fold are the extended loops shown for BAG1 and UBL7, to the right hand side of molecular tube plots shown (panel C, lower section). These loops are also apparent as the biggest differences in the structure-based sequence alignment. Although BAG1 and UBL7, with an extended loop, do not bind SGTA, neither does ubiquitin, which lacks an extended loop. The alignment is arranged with the three non-SGTA binders above the three SGTA binders. Whilst there is no consistent localized change in sequence that clearly separates these two sub-groups, there is a difference in the net charge predicted at neutral pH and summed over the aligned sequences. Hence for non-SGTA binders these values are −1, −2 and −1 respectively, whilst for the SGTA-binders these values are +2, +5 and +2 (order of net charge values as per the alignment). Color coded molecular surfaces (blue = basic and red = acidic) are shown to illustrate the most extreme difference, UBL4A (+5) and BAG1 (−2) (panel C, lower section), suggesting that a difference in charge presentation to SGTA may contribute to binding specificity. Recent high-resolution structures of an equivalent region of the homologous Sgt2-Get5 complex [22], [23] indicate that this effect is likely to be more extensive than direct charge complementarity at the interface between a dimeric form of the SGTA N-terminus and its partner UBL [24].

Given the structural similarities between UBLs and ubiquitin, we investigated the possibility that SGTA might bind mono-ubiquitin. Although radiolabelled mono-ubiquitin was efficiently synthesized in the cell free system, we saw no evidence of salt sensitive binding to immobilized SGTA (Figure 4A, cf. lanes 2 and 5, see arrow). In contrast *in vitro* synthesized ubiquitin bound to the immobilized UBA domain from Dsk2, a known ubiquitin binding protein under conditions where no binding to SGTA was detected (Figure 4B, cf. lanes 3 to 6). This interaction confirms that *in vitro* synthesized ubiquitin is correctly folded. UBL4A was efficiently bound to SGTA, but any interaction with the Dsk2-UBA did not appear to be above background (Figure 4B, cf. lanes 10 to 13). Thus, amongst the substrates tested, we find that SGTA binds to subunits of the BAG6 complex that bear a UBL (BAG6 and UBL4A), and to a conserved yeast equivalent (Get5), but not to other UBL domain containing proteins (UBL7 and BAG1) or ubiquitin. Interestingly, in the Dsk2-UBA pull downs we observe preferential binding of higher molecular weight species that we speculate may be polyubiquitin chains generated in the cell free translation system (Figure 4B, lane 4, filled circles).

### Recapitulating SGTA-UBL Binding with Recombinant Proteins

**Figure 5:**
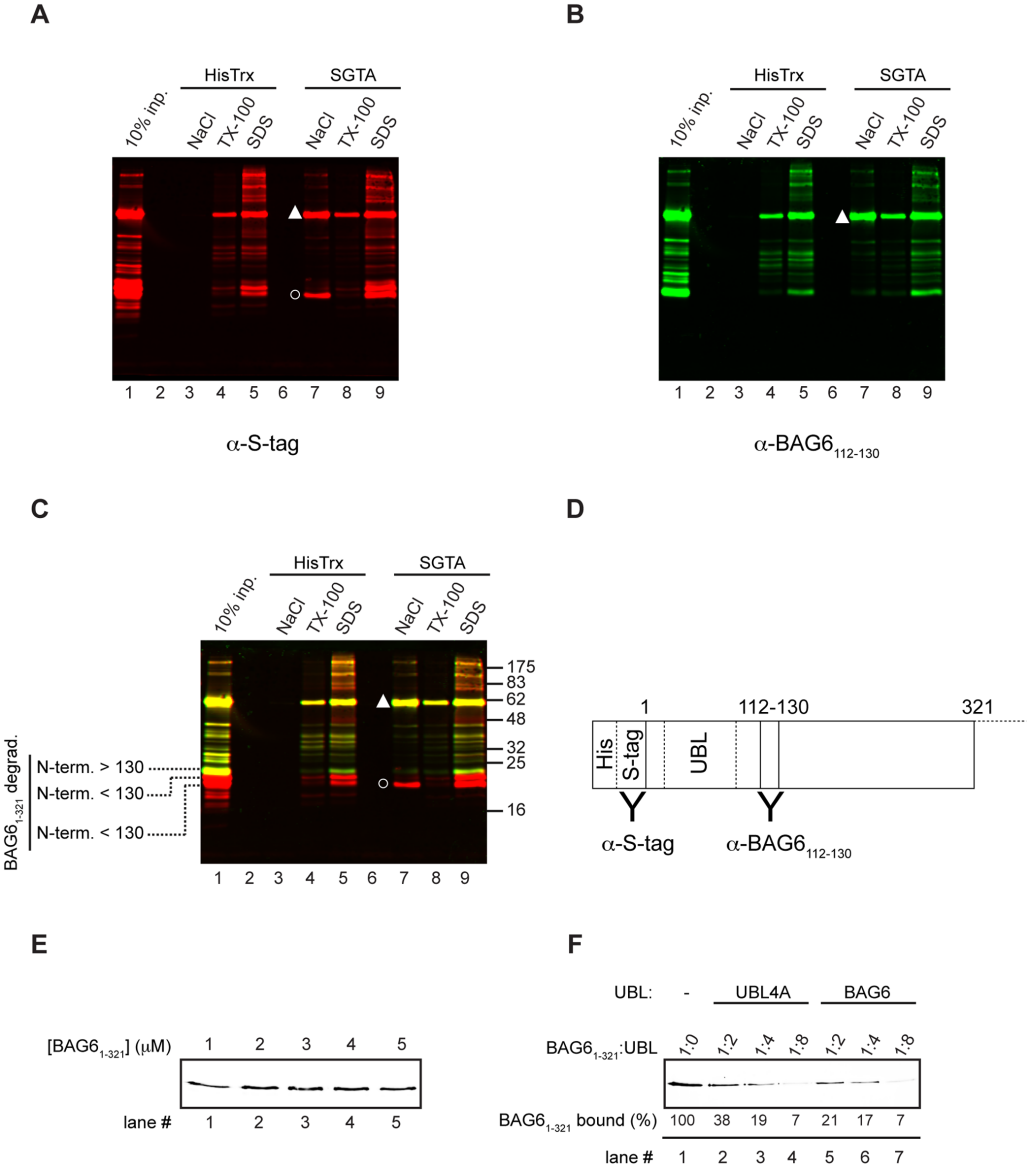
A recombinant BAG6 fragment binds SGTA via its UBL. A recombinant form of human BAG6 (isoform 2) encoding residues 1 to 321 and bearing N-terminal polyhistidine- and S-tags was incubated with immobilized HisTrx and SGTA and processed as previously described, together with a fraction of the input (equivalent to 10% of the amount added to the pull down assay). In this case bound material was resolved by SDS-PAGE and analyzed by immunoblotting with antibodies recognizing the S-tag (A) and a BAG6 derived peptide (B) that were discriminated using different secondary antibodies. Salt sensitive binding of the recombinant BAG6 fragment (BAG6_1–321_) to SGTA is indicated by a filled triangle (see panels A and B, lane 7), and the presence of both antibody epitopes was confirmed in the merged image where the product is yellow (panel C, lane 7, see filled triangle). The recombinant protein contained a number of BAG6 derived degradation products (BAG6_1–321_degrad. see also Figure S4). One such product displays enhanced binding to SGTA (panel A, lane 7, open circle) but lacks the BAG6 derived epitope as indicated by the merged image (panel C, lane 7, open circle, product labeled in red channel only). The location of the S-tag and the BAG6 epitope are shown in schematic form (D). The binding capacity of a fixed amount of SGTA coupled beads was determined empirically by adding increasing amounts of recombinant BAG6_1–321_ and found to be saturated at a final concentration of 2 µM (E). A competition experiment (F) was performed by incubating the same amount of SGTA coupled beads and 2 µM BAG6_1–321_ as before (lane 1), or with increasing amounts of recombinant UBLs derived from UBL4A (lanes 2 to 4) or BAG6 (lanes 5 to 7). The amount of BAG6_1–321_ recovered in each case was estimated by quantitative immunoblotting and expressed as a percentage of the recovery obtained in the absence of any competing UBL (lane 1). The estimated molar ratio of BAG6_1–321_ to recombinant UBL for each reaction is indicated, and an independent repeat of this experiment is shown in the Figure S5).

Having defined an N-terminal region of BAG6 as essential for its interaction with SGTA we wished to exclude the possibility that, even using a wheat-germ translation system, endogenous components might contribute to the binding detected (cf. Figures 1 to 3). To this end we sought to recapitulate the interaction using purified recombinant components. An N-terminal fragment of BAG6 that is slightly bigger than the 270-residue fragment analyzed *in vitro* (cf. Figures 1 and 2) was successfully expressed in *E. coli* and purified (Figure S4). This recombinant form of BAG6 includes N-terminal poly-histidine and S-tags fused in frame to the first 321 residues of the coding region (His-S-BAG6_1–321_), and it was tested for SGTA binding. Recombinant His-S-BAG6_1–321_ displays robust, salt sensitive, binding to immobilized SGTA, but not to a thioredoxin control (Figures 5A to 5C cf. lanes 3 and 7, filled triangle). Three shorter N-terminal degradation products (denoted BAG6_1–321_degrad.) co-purify with His-S-BAG6_1–321_ (cf. Figure S4), and one of these products displays strongly enhanced binding to immobilized SGTA (Figure 5A, lane 7, open circle). By using an epitope specific antibody (see Figure 5D) we were able to confirm that this particular BAG6 derived fragment has an intact N-terminal S-tag but terminates before residue 130 of the BAG6 coding region (see Figures 5C and 5D), and hence establish that it incorporates the entire UBL region of the BAG6 N-terminus.

SGTA and its yeast equivalent, Sgt2, appear to from stable dimers [34], [36]. Having shown that immobilized SGTA can bind the UBLs of two distinct subunits of the BAG6 complex, i.e. BAG6 and UBL4A, we asked whether the UBLs of both components can bind simultaneously to SGTA. Having established conditions where His-S-BAG6_1–321_ was present in excess over the binding capacity of immobilized SGTA (see Figure 5E), we carried out competition experiments in the presence of increasing amounts of recombinant UBLs derived from either UBL4A or BAG6, and found that both UBLs competed effectively for His-S-BAG6_1–321_ binding to SGTA (see Figure 5F and Figure 5). These data support a model where the N-terminal UBL of BAG6 plays a vital role in its interaction with SGTA, and suggest that an SGTA dimer can accommodate either one copy of BAG6, or one copy of UBL4A, but not both at the same time (see Discussion).

### The UBL and BAG Domains of BAG6 are Dispensable for Substrate Binding

**Figure 6:**
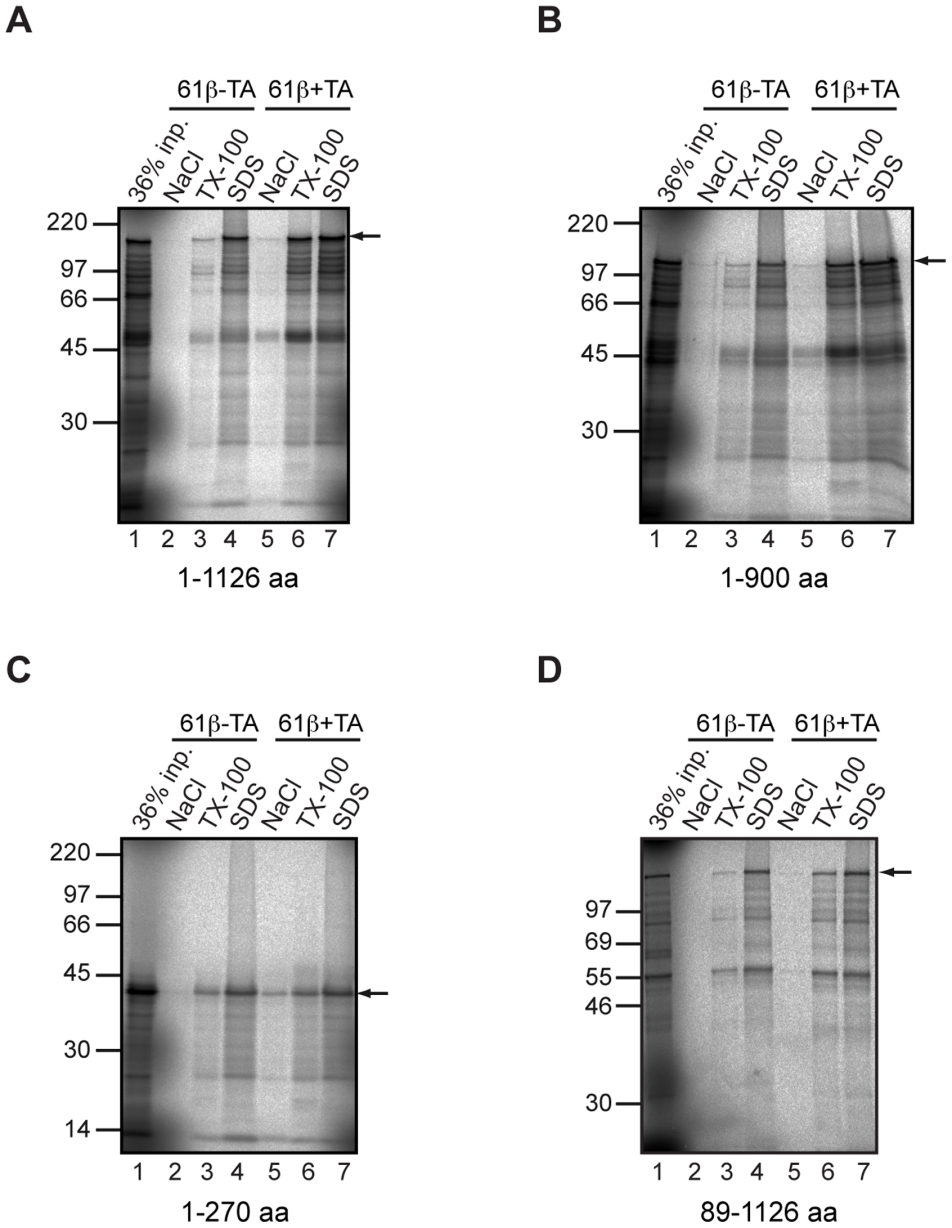
The binding of BAG6 deletion mutants to an immobilized TA protein. Radiolabelled full-length BAG6 (A) and fragments lacking the C-terminal 226 residues including the BAG domain (B), encoding the N-terminal 270 residues only (C) or lacking the N-terminal UBL (D) were synthesized *in vitro* as before and incubated with immobilized recombinant Sec61β with an intact (+TA) or deleted (−TA) tail-anchor region. Samples were processed as described for Figure 1 and bound material analyzed by SDS-PAGE and phosphorimaging. In this case BAG6 binding was sensitive to Triton-X100, and an arrow indicates the location of the relevant BAG6 derived product (cf. signals in lanes 3 and 6 in each panel). With full length BAG6, quantification showed that the signal obtained with the control protein lacking the TA region (Sec61β−TA) was less than 3% of that recovered using Sec61β with an intact tail-anchor (Sec61β+TA).

In an effort to delineate the substrate-binding site of BAG6 we altered the bait in our pull-down assays to recombinantly expressed Sec61β, a well-defined TA protein substrate of BAG6 [7], [14], [15]. Since the interaction of TA proteins with BAG6 relies on the TA region [14], [15], we used a version of Sec61β lacking the TA as a control for binding specificity. Full length BAG6 binds efficiently to immobilized Sec61β bearing an intact hydrophobic tail-anchor region (Sec61β+TA), and this association is resistant to the salt elution step that releases it from SGTA (Figure 6A, cf. Figures 1 and 2). Rather, BAG6 is specifically released from immobilized (Sec61β+TA) by treatment with the non-ionic detergent Triton X100 (Figure 6A, lane 6, see arrow), consistent with an interaction based on hydrophobic contacts [7], [8], [14]. In contrast, when a form of immobilized Sec61β that lacks a tail-anchor region (Sec61β-TA) is used, only a weak background signal is observed, confirming the specificity of the interaction (Figure 6A, cf. lanes 3 and 6, arrow).

**Figure 7:**
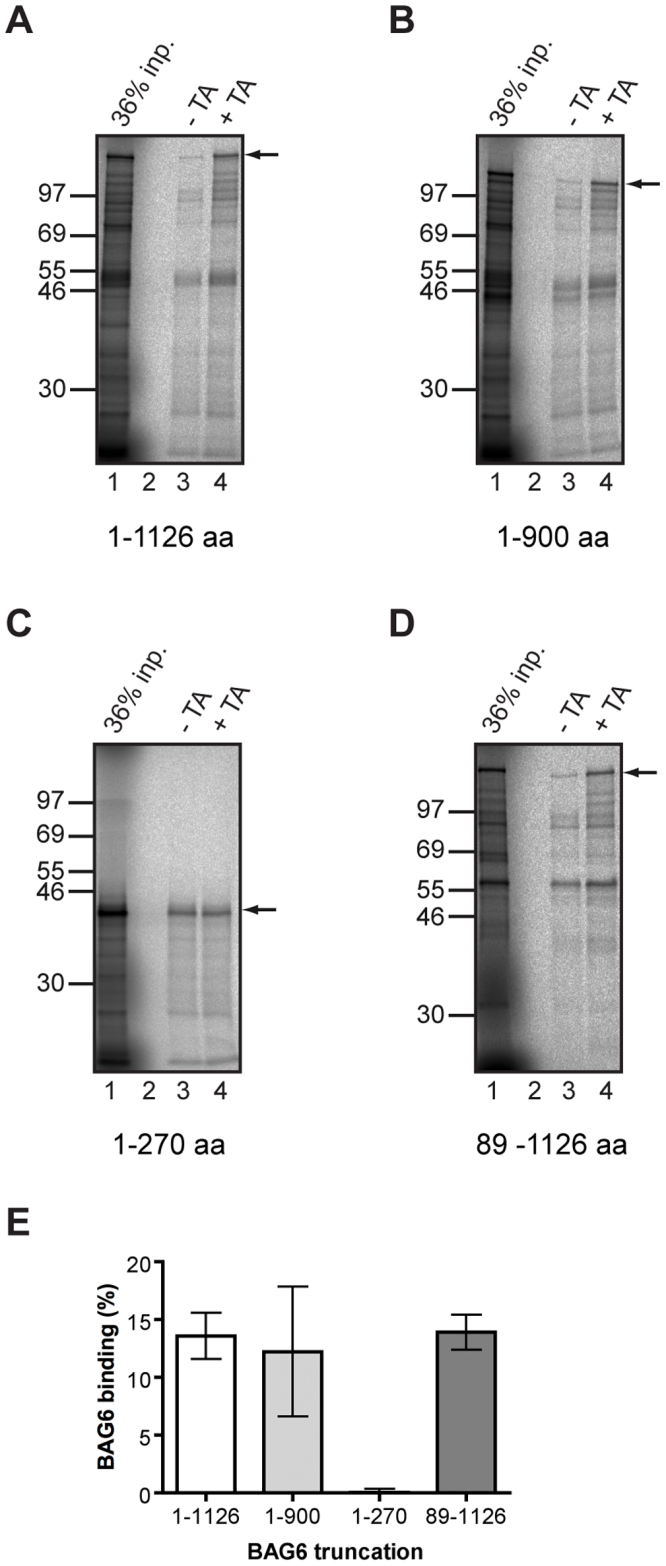
The UBL and C-terminus of BAG6 are dispensable for binding a TA protein. The Triton-X100 sensitive binding of full-length BAG6 (A) and three deletion mutants (B to D) to a TA region was quantified using phosphorimaging to measure the appropriate translation product (see arrows). In each case the signal recovered using immobilized, recombinant Sec61β lacking an intact TA (−TA, see lane 3 for each panel) was compared to that using recombinant Sec61β bearing the TA (+TA, see lane 4 for each panel) and the former value subtracted from the latter to remove any potential contribution by TA independent interactions. The resulting value was expressed as a percentage of the input fraction (see lane 1 for each panel). The numbers presented in the bar graph are each derived from at least three independent experiments (n = 3) and indicate the standard deviation.

We next investigated the importance of the BAG6 UBL and BAG domains for its ability to bind to the Sec61β TA protein substrate. The N-terminal 900 residues of BAG6 displayed a robust Triton sensitive interaction qualitatively comparable to the wild-type protein (Figure 6B, cf. lanes 3 and 6, arrow). However, the N-terminal 270 residues of BAG6, which performed well in the SGTA binding assay (Figures 1 and 2), showed no apparent increase in binding to the same immobilized substrate bearing an intact TA region when compared to its matched control (Figure 6C, cf. lanes 3 and 6, arrow). Removal of the N-terminal UBL from BAG6 did not affect TA protein binding as compared to the full-length protein (Figure 6D, cf. lanes 3 and 6, arrow). In order to elaborate on the effects of these various deletions, we repeated these binding experiments under conditions where the Triton sensitive material bound to versions of recombinant Sec61β with and without a TA could be directly compared and quantified. In each case, the signal recovered in the control, Sec61β-TA, sample was used as a measure of background/non-specific binding and subtracted from the signal recovered using immobilized Sec61β with an intact TA region (Figures 7A to D). The resulting values provide a measure of specific, i.e. tail-anchor dependent, binding. This analysis showed that the loss of either the N-terminal UBL (BAG6 89–1126) or the C-terminal BAG domain (BAG6 1–900) had no quantitative effect on binding when compared to full-length wild type BAG6 (Figure 7E). In contrast, the N-terminal 270 residues of BAG6 are incapable of specific binding to an immobilized TA protein substrate (Figure 7E).

**Figure 8:**
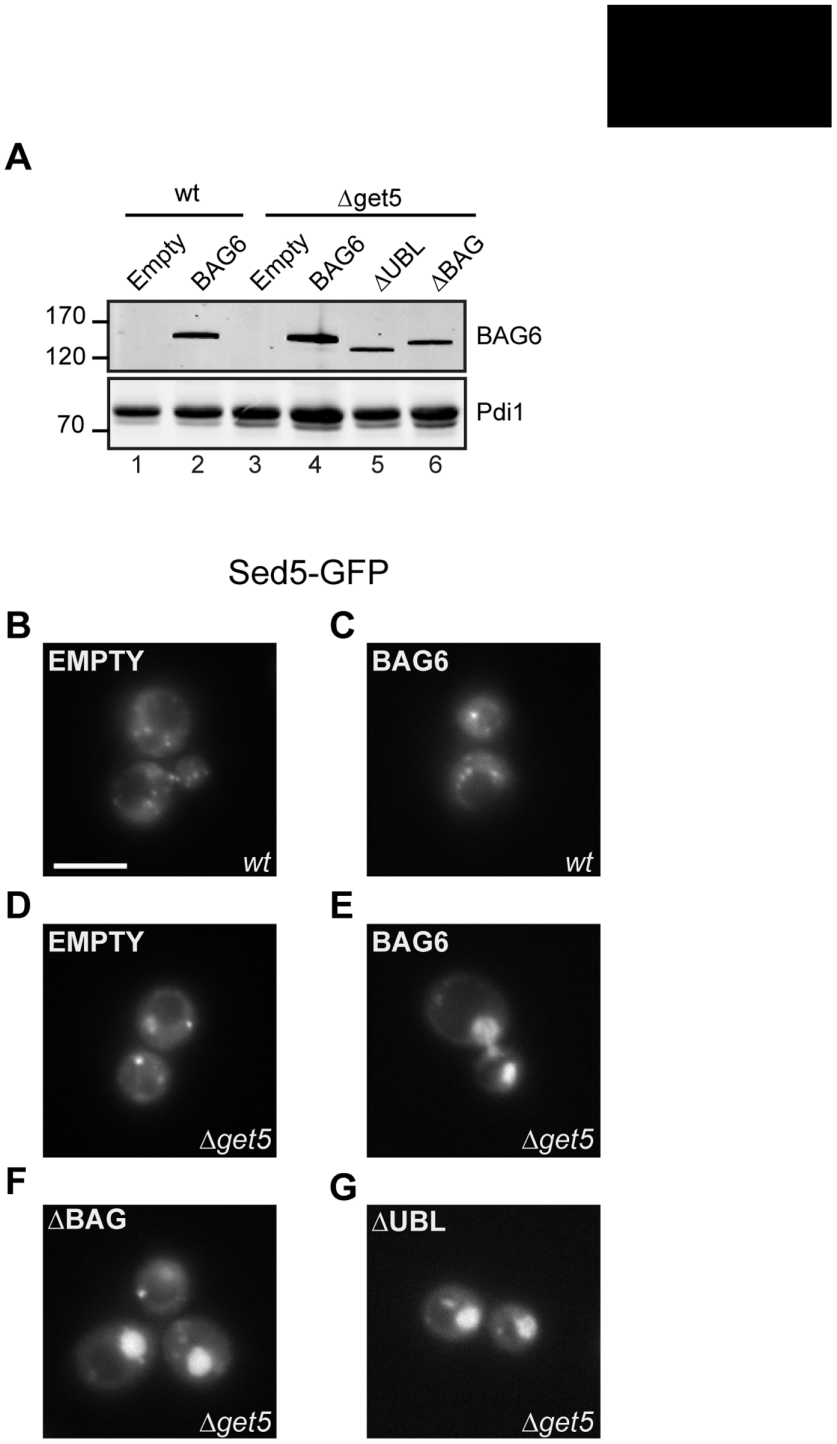
UBL and BAG domains are dispensable for BAG6 mediated relocalization in yeast. A wild type (wt) or *Δget5 (Δmdy2)* strain was transformed with a plasmid encoding GFP-Sed5 together with a second plasmid encoding: full-length BAG6 (BAG6), residues 1 to 1050 of BAG6 (ΔBAG) or residues 89 to 1126 of BAG6 (ΔUBL) as indicated. Alternatively, the p416Met25 plasmid alone was used (EMPTY). Total cell lysates were prepared with samples normalized to the optical density of the cultures, and levels of the BAG6 variants determined by immunoblotting (Panel A, see BAG6). The levels of protein disulfide isomerase were used as a loading control (Panel A, see Pdi1). The subcellular localization of GFP-Sed5, and impact of co-expressing BAG6 or its derivatives upon its location, was determined by live cell imaging of wild type (wt, panels B and C) and *Δget5* cells (panels D to G) as indicated. Scale Bar = 5 µM.

In a previous study we established a novel substrate relocalization assay that uses the heterologous expression of mammalian BAG6 in *S. cerevisiae* GET (Guided Entry of TA proteins) pathway mutants that are defective in TA protein biogenesis [14]. Strikingly, when BAG6 is expressed in such strains together with a GFP-tagged TA-protein, the GFP-Sed5 is relocalized to the nucleus. This relocalization of GFP-Sed5 requires that a naturally occurring nuclear localization signal that is present in BAG6 remains intact (cf. Figure 1A), and does not rely on the expression of any of the known soluble components of the *S. cerevisiae* GET pathway [14]. These data support a model where BAG6 interacts directly with TA-protein substrates, even in the heterologous environment of a yeast cell [14], and we used this assay as an independent approach to investigate the binding of BAG6 mutants lacking the UBL (ΔUBL = BAG6, residues 89–1126) or the BAG domain (ΔBAG = BAG6, residues1–1050) to GFP-Sed5, a second tail-anchored membrane protein substrate. The expression of full-length wild-type BAG6 (cf. Figure 1A), and the two deletion mutants was analyzed by immunoblotting and we found all three proteins were expressed in a *Δget5* strain lacking a functional GET pathway (Figure 8A, cf. lanes 4 to 6). Full-length BAG6 was also efficiently expressed in a wild type yeast strain (Figure 8A, cf. lanes 1 and 2). We next analyzed the effect of heterologous BAG6 expression upon GFP-Sed5, and found it to have no effect on its subcellular localization in a wild-type strain with a functional GET pathway (Figure 8, cf. panels B and C) as previously reported [14]. In contrast, the expression of full-length BAG6 in the *Δget5* strain resulted in a striking relocalization of GFP-Sed5 to the nucleus (cf. Figures 8D and 8E) consistent with our previous study [14]. Likewise, the expression of the two BAG6 mutants lacking either the BAG or UBL domain also resulted in the relocalization of GFP-Sed5 to the nucleus (cf. Figures 8D, 8F and 8G). These data are in full agreement with our biochemical analysis of BAG6 binding to immobilized Sec61β, and taken together suggest that both the UBL and BAG domains of the BAG6 protein play no role in its ability to bind to TA protein substrates either *in vitro* or in a heterologous yeast expression system.

## Discussion

A role for BAG6 in the recognition and removal of hydrophobic substrates from the cytosol was first apparent when it was identified as part of an upstream loading complex for the TRC40 dependent delivery of TA proteins to the ER membrane [14], [15], [18]. However, it is now clear that BAG6 substrates are not restricted to TA proteins on a productive pathway for ER delivery, and it can also participate in the ubiquitin dependent degradation of a range of precursor proteins that fail to be delivered to the ER and misfolded proteins that have been retrotranslocated from the ER [32].

### UBL Mediated Recruitment of SGTA to BAG6 and UBL4A

**Figure 9:**
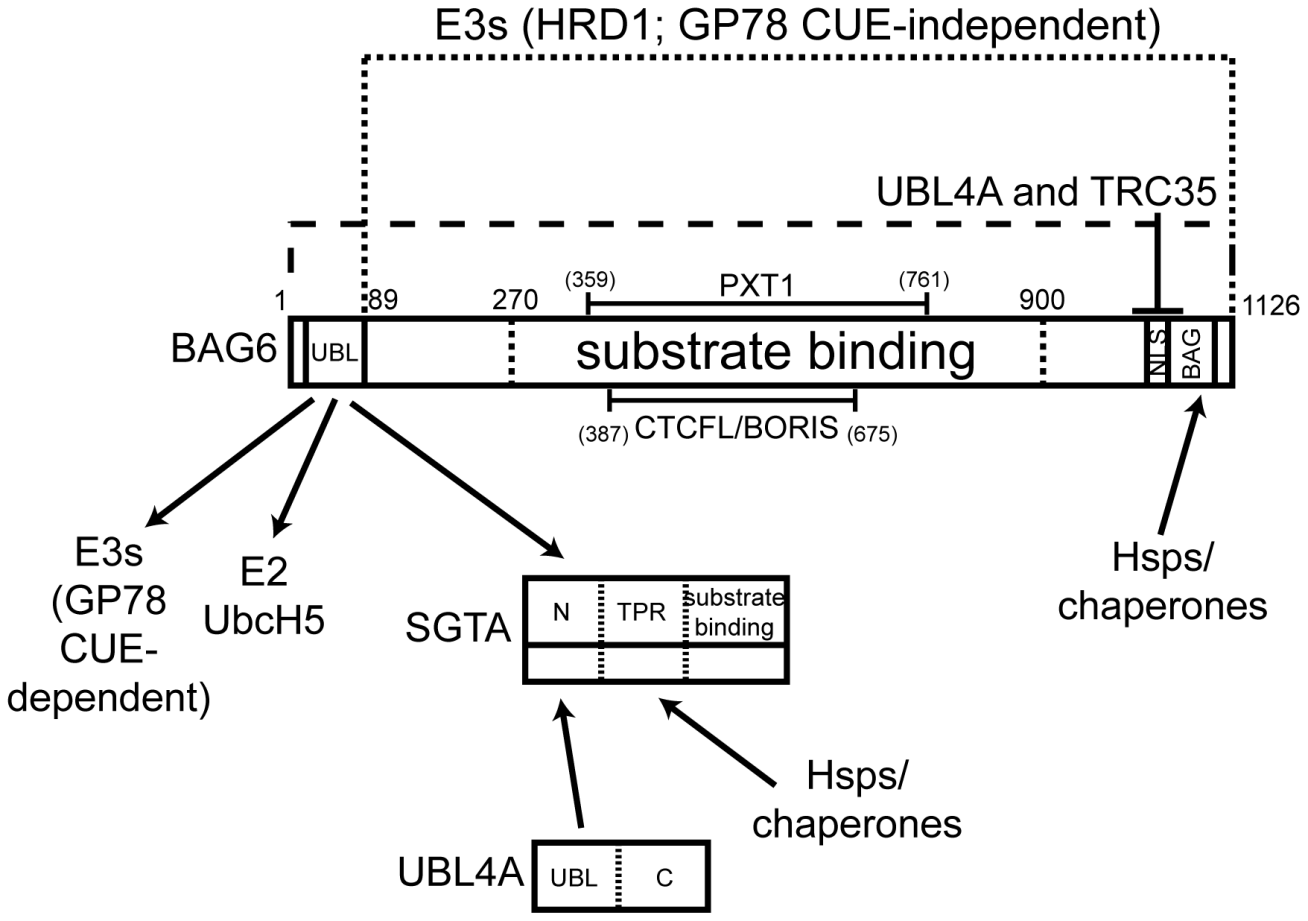
Model summarizing BAG6 interactions with putative substrates and effectors. The results of this study identify an interaction of the N-terminal UBL of BAG6 with SGTA, and the hydrophobic region of TA proteins with a region located between residues 90 and 900 where residues 1 to 270 alone are incapable of interacting *in vitro*. Published studies have identified the following interactions/binding partners: the N-terminal UBL of BAG6 is necessary for both the UbcH5 dependent ubiquitination of mislocalized proteins [30] and its binding to the CUE domain of the E3 ligase GP78 [29]; the N-terminal 471 residues are sufficient to associate with polyubiquitinated substrates [27]; a region located between residues 387 and 675 provides the site of BAG6 binding to CTCFL/BORIS [40] whilst an overlapping region located between residues 359 and 761 appears to mediate the interaction between BAG6 and PXT1 [41]. The precise assembly of BAG6 with UBL4A and TRC35 remains to be defined, but TRC35 alone appears to be sufficient to prevent BAG6 import into the nucleus, potentially by masking its NLS [29]. BAG domains mediate direct interactions with the nucleotide-binding domain of Hsp70 chaperones [49]. We have also identified a direct interaction between the UBL of UBL4A and SGTA and propose that this represents an association with a dimer of SGTA [22], [23], [24], [34]. The role of the C-terminal region of SGTA, and its yeast ortholog Sgt2, in binding hydrophobic substrates is well established [17], [36]. TPR (tetratricopeptide repeat) domains act as sites of interaction with a range of components including Hsp70s, Hsp90s and other chaperones [50]. In yeast Sgt2, the equivalent region appears to be dispensable for several of its known functions [51]. Likewise the loss of the canonical TPR domains from mammalian SGTA does not perturb its ability to antagonize BAG6 function and promote the deubiquitination of mislocalized proteins [31].

Previous studies identified mammalian SGTA as an interacting partner of BAG6 [19], [20], and we now show that the N-terminal UBL of BAG6 is essential for its *in vitro* binding to SGTA. Hence, the deletion of the UBL from *in vitro* synthesized BAG6 fragments results in the loss of SGTA binding (Figures 1 and 2), whilst a truncated recombinant fragment of the BAG6 N-terminus with an intact UBL-region (Figures 5A to 5C), and the BAG6-UBL alone (Figure 5E), are both able to bind to SGTA. Our *in vitro* binding studies suggest that longer BAG6 fragments may bind more effectively to SGTA (cf. Figure 2). Whilst this could reflect a contribution from additional elements C-terminal of the UBL [37], it may equally reflect enhanced folding of the UBL domain when present in the context of longer BAG6 fragments. Based on these data we conclude that SGTA is one of several potential effectors that can bind to BAG6 via its N-terminal UBL (cf. Fig 9).

We also observe a robust interaction between UBL4A and SGTA, and find this binding to be mediated by the N-terminal UBL of UBL4A, consistent with several recent structural studies [22], [23], [24]. However, unlike Sgt2, SGTA has at least two potential UBL mediated interactions with components of the equivalent upstream loading complex for TA protein biogenesis, i.e. BAG6 and UBL4A. SGTA is not however a generic binding partner of UBL domains, and we see no evidence for any interaction with two unrelated UBL-containing proteins or ubiquitin. A structure-based alignment of these different domains (see Figure 4C) suggests that differences in their net charge presentation contribute to their ability to bind SGTA (see also Legend to Figure 4C). This is borne out by recent high-resolution structural studies of the N-terminal dimerization region of SGTA/Sgt2 in complex with the UBL of UBL4A/Get5 that reveal strong electrostatic interactions between the two components [22], [23], [24], and provide a molecular basis for the salt sensitive binding observed between them (this study; see also [24]). There is now compelling evidence that a dimer of SGTA/Sgt2 binds to a single UBL domain [22], [23], [24], [34], and our data strongly support this stoichiometry for the interaction between SGTA and its two potential UBL-bearing partners, BAG6 and UBL4A. Specifically, UBLs derived from both UBL4A and BAG6 each compete with His-S-BAG6_1–321_ for SGTA binding, effectively ruling out a scenario where two UBLs from different proteins can both be accommodated together by SGTA. This model is also supported by our observation that the UBL from *S. cerevisiae* Get5 binds well to mammalian SGTA, further suggesting structural conservation of the interaction. We therefore conclude that a dimer of SGTA can bind either BAG6, or UBL4A, but not both components at the same time.

Paradoxically, whilst SGTA can bind to BAG6 (this study; [19], [20]), UBL4A (this study; [24]) and TA proteins [14], [25], it does not appear to behave as a stable component of the BAG6-complex [15], [29], [30]. This is also consistent with studies of the *S. cerevisiae* upstream loading complex, which show the association between Get5 and Sgt2 is labile and/or transient in nature [33], [34]. Taken together, our data are consistent with a model where a variety of different effectors, including SGTA, can compete for binding to the UBL domain of BAG6 in order to access bound substrates (see Fig 9). It is plausible that the binding of SGTA to the BAG6-UBL may be influenced, or attenuated, by competition for binding to the UBL4A-UBL, or vice-versa, and it has been suggested that UBL4A may be a preferential partner for the SGTA N-terminal dimerization region in vivo [24].

### Substrate Binding to BAG6

The binding of the BAG6-complex to hydrophobic substrates including TA proteins, mislocalized proteins and ERAD substrates, potentially reflects the actions of up to three subunits [15], [29], [38]. Our previous work suggested a direct interaction between the BAG6 subunit and TA protein substrates [14], and the data presented here fully support such a model. Specifically, we developed an *in vitro* binding assay to determine the contribution of the UBL and BAG domains of BAG6 to its interaction with a model TA protein, Sec61β, and found that the loss of either had no effect. Likewise, using Sed5-GFP as a second model TA protein and analyzing its interaction with BAG6 via a yeast based nuclear relocalization assay, we also find no evidence of a role for the UBL or BAG domains in substrate binding. The binding of full-length BAG6, and its deletion mutants, to immobilized Sec61β is characterized by its sensitivity to Triton X-100, a phenomenon that most likely reflects the hydrophobic nature of the interaction [7], [8], [14]. A short N-terminal 270 residue fragment of BAG6 is unable to bind an immobilized TA protein despite its robust interaction with SGTA. Our data suggest that the binding of BAG6 to hydrophobic substrates most likely involves one or more elements present in the large central region between the N-terminal UBL and the C-terminal BAG domain, i.e. between residues 90 and 900, and suggest that residues 90 to 270 alone are incapable of interacting with such substrates.

The central region of BAG6 is proline-rich and has repeated domains, but is otherwise poorly defined [39]. Interestingly, binding sites for CTCFL/BORIS [40] and Ptx1 [41] are also located within this portion of BAG6 (cf. Figure 9). Furthermore, several studies have linked BAG6 to distinct complexes that each mediates some form of post-translational modification, namely ubiquitination [29], [30], methylation [40] and acetylation [42], [43]. Thus, BAG6 may act as a scaffold that orchestrates the presentation of various “substrates” to a range of cofactors and effectors that can modify their lysine side chains. Our data also provide a rationale for the previously reported dominant negative effect of ΔUBL-BAG6 on protein degradation [30]. Hence, this truncated form of BAG6 would bind hydrophobic substrates but be unable to recruit any effectors that require the UBL, thereby protecting bound precursors from ubiquitination and degradation (cf. Figure 9).

Although the BAG6 protein is named on the basis of its BAG domain, we were unable to detect any contribution of this region with the assays at our disposal, and our studies provide no new insights into its role in the context of BAG6. In the case of the BAG6-UBL we clearly establish that it plays a key role in the association of the BAG6 protein, and hence presumably the BAG6 complex, with SGTA. Likewise the UBL domain of the UBL4A subunit of the BAG6 complex can also bind SGTA, and hence there are two potential sites of interaction for SGTA and the BAG6 complex. In addition to its ability to bind to the UBLs present on the BAG6 complex, SGTA also has proven capacity to bind to hydrophobic substrates [25], [36], and can influence BAG6 mediated quality control. Firstly, SGTA may act upstream of BAG6 to promote the binding of misfolded proteins on a pathway for ER associated degradation [24] and, secondly, SGTA can antagonize the actions of the BAG6 complex by promoting the deubiquitination of its mislocalized membrane protein substrates, thereby inhibiting their proteasomal degradation [31]. Whether a direct interaction between the BAG6 complex and SGTA is important for this “rescue” of ubiquitinated BAG6 substrates is unclear. However, it is possible that there is competition for binding to the UBLs of the BAG6 complex between factors that promote ubiquitination (E2 and E3 enzymes), and those that promote deubiquitination (SGTA and associated deubiqitinases). Such a dynamic system would provide a basis by which the fate of hydrophobic substrates that are bound to the BAG6 complex can be carefully regulated, and is consistent with the operation of a BAG6/SGTA cycle for protein quality control in the cytosol [31].

## Materials and Methods

### Antibodies and Plasmids

The mouse monoclonal antibody recognizing the S-tag epitope was purchased from Novagen, whilst a rabbit polyclonal serum recognizing residues 112–130 of BAG6 isoform 2 was made to order by Peptide Specialty Laboratories GmbH. The cDNAs coding for BAG6 isoform 2 and UBL7 were purchased from Origene, whilst UBL4A was from GenScript and BAG1 was a gift from Dr. Doug Cyr (University of North Carolina, USA). Where necessary, the coding regions were subcloned into vectors suitable for *in vitro* transcription. Yeast expression constructs coding for BAG6 full-length protein or its truncations were prepared as described previously [14]. BAG6 DNA fragment corresponding to residues 1–321 of isoform 2 was cloned into pET30a bacterial expression vector (Novagen) with in frame 6His- and S-tags at the 5′ end using EcoRI and XhoI restriction sites.

### Recombinant Protein Production

Sec61βOPG was purified as described previously [14]. HisTrx, HisTrx-SGTA and the His-S-BAG6_1–321_ fragment were purified essentially in the same way as Sec61βOPG [14] but bacteria were lysed by sonication rather than detergent lysis, and proteins were eluted from NiNTA agarose (Qiagen) in a step-wise manner in the presence of increasing imidazole concentrations. Buffer from the eluted fractions was exchanged into PBS and protein concentration determined by densitometric analysis using BSA as a standard. The UBL domains of UBL4A (1–74) and BAG6 (1–101) were cloned into the pET-46 vector and the resulting plasmids were used to transformed *E. coli* Rosetta cells which were then grown to OD_600_ = 0.8, induced with 0.5 mM IPTG, and the proteins expressed overnight at 30°C. After cell lysis by sonication, the recombinant UBLs were purified by affinity chromatography using HisPur™ Cobalt Resin (Thermo Scientific). These two polypeptides are UV-invisible, hence their concentrations were estimated by comparing isolated methyl peaks with those of a known concentration of reference compound 2,2-Dimethyl-2-silapentane-5-sulfonate (Sigma Aldrich) in 1D NMR spectra.

### In vitro Transcription, Translation and Pull-down Assays

Templates for *in vitro* transcription reactions were generated by PCR using appropriate primers and RNA prepared as previously described [3]. Proteins were translated using a wheat-germ system (Promega) for 1 h at 25°C in the presence of 1 mCi/ml [^35^S]methionine. Translations were terminated by the addition of puromycin to a final concentration of 1 mM and further incubation for 10 min at 25°C. RNaseA was then added (0.5 mg/ml), reactions incubated for 5 min at 37°C and any particulates sedimented by centrifugation for 2 min at 9,500×g. Pull-down assays were carried out by mixing 22 µl of such translation reactions with 5 µl of the indicated proteins previously immobilized on UltraLink Biosupport according to manufacturer’s instruction [see also [14]]. Reactions were incubated for 2 h at 25°C with constant agitation; beads were extensively washed with buffer R (50 mM HEPES-KOH, pH 7.5, 40 mM potassium acetate, 5 mM MgCl_2_) and the bound material sequentially eluted with buffer R supplemented with 1 M NaCl, buffer R supplemented with 0.5% (v/v) Triton X-100 and, finally, with SDS sample buffer. Samples were resolved by SDS-PAGE and results visualized by phosphorimaging using Fuji BAS 3000 PhosphorImager system (Fuji Photo Film, Tokyo, Japan). When analyzing the binding of radiolabelled substrates to immobilized Dsk2-UBA, we followed the manufacturers instructions (Enzo Life Sciences), employing the same conditions in parallel for analyzing binding to SGTA (see Figure 4B).

Pull-down reactions with purified components were carried out in the same manner using 2 µM His-S-BAG6_1–321_. Samples were resolved by SDS-PAGE, transferred onto PVDF membrane and proteins detected using quantitative immunoblotting [25]. Prior to the UBL competition experiment, the amount of BAG6_1–321_ required to saturate the SGTA coupled beads was determined by incubating a fixed volume of beads with 1 to 5 µM His-S-BAG6_1–321_ for 1 hour at 25°C. The resin was washed repeatedly with buffer R before eluting the bound material using buffer R containing 1M NaCl. To determine whether the UBL domains of either UBL4A or BAG6 could compete with His-S-BAG6_1–321_ for SGTA binding, the UBLs were pre-incubated with the pre-determined amount of SGTA-coupled beads for 1 hour at 25°C in buffer R, and then 2 µM His-S-BAG6_1–321_ was added to give ratios of 1∶0, 1∶2, 1∶4 and 1∶8 relative to the competing UBLs from UBL4A and BAG6. After a further 1 hour incubation the resin was washed, and following elution the amount of bound BAG6 was quantified by SDS-PAGE and immunoblotting.

### Analysis of BAG6 Truncations in a Yeast GET Mutant

BAG6-derived constructs, i.e. variants lacking either the N-terminal UBL or the C-terminal BAG domain were expressed from p416Met25 as previously described [14]. GFP-Sed5 was expressed from plasmid pRS413, a construct obtained by the subcloning of GFP-Sed5 from pRS315 Sed5-GFP [44]. All yeast strains used were derived from BY4741 (MATa *his3Δ1 leu2Δ0 met15Δ0 ura3Δ0*) [45]. The deletion strain for *GET5/MDY2* (*Δget5/mdy2::Kan^R^*) was obtained from Euroscarf [46]. Yeast transformation and growth in synthetic complete media lacking uracil and histidine and preparation of total cell lysates for immunoblotting analysis followed well-established protocols [47], [48]. The rabbit anti-BAG6 and anti-PDI antibodies were both used at 1∶1,000 and secondary antibodies (Li-Cor) at 1∶10,000. Live cell imaging of yeast cells was performed at room temperature in synthetic complete medium employing a DeltaVison restoration microscope equipped with a 100×/1.4 UplanSApo objective and a GFP filter set (475/28 nm). The images were collected with a Coolsnap HQ camera (Photometrics).

## Supporting Information

Figure S1 **BAG6 associates with SGTA in a salt-sensitive manner.** BAG6 fragments encompassing residues 1 to 270 (A) or 500 to 1126 (B) were synthesised *in vitro* using a wheat-germ extract and incubated with immobilized BSA or SGTA as shown (see Materials and Methods). Unbound material was collected, beads washed five times with low-salt buffer, followed by elution with buffer containing 1M NaCl, 0.5% (v/v) Triton X-100 and finally with SDS-PAGE sample buffer. A fraction of input, equivalent to 36% of the material used for the binding reaction, the unbound material, low salt washes 1 and 5 and the material eluted with NaCl, Triton X-100 and SDS-PAGE sample buffer were resolved by SDS-PAGE and the products visualized by phosphorimaging. An arrow indicates the location of the relevant translation product in each of the reactions. The same exposure of a single gel is shown with irrelevant lanes removed for clarity. As can be seen, bound material is eluted from immobilized SGTA with NaCl when the BAG6 fragment contains an intact N-terminal region (panel A, cf. lanes 5 and 12), but not when the N-terminal regions is absent (panel B, cf. lanes 5 and 12).
(TIF)

Figure S2 **BAG6 interaction with SGTA is highly reproducible.** Additional examples of the binding of full length BAG6, and BAG6 fragments, to immobilized BSA and SGTA are shown. These represent independent repeats of the qualitative pull down experiments presented in Figure 1 of the main text, and illustrate the highly reproducible nature of the salt sensitive interaction between fragments of BAG6 with an intact N-terminal UBL and SGTA.
(TIF)

Figure S3 **Analysis of SGTA interactions using additional BAG6 fragments and the UBL domain from** ***S. cerevisiae*** **GET5.** Additional N-terminal fragments of BAG6 (A–D) that are not presented in the main text, and the UBL domain from *S. cerevisiae* GET5, Sc_Get5-UBL, (E; cf. main text, Fig. 3C) were translated *in vitro* using wheat-germ extract and their binding to immobilized BSA and SGTA analyzed as described for Figure 1 of the main text (see also Materials and Methods). We consistently observe that N-terminal fragments of BAG6 that contain up to 700 residues show an abnormal migration on SDS-PAGE, a behavior that most likely reflects the comparatively high proportion of proline residues located in this region of the protein.
(TIF)

Figure S4 **Purification of His-S-BAG6_1–321_.** N-terminal 1–321 residues of BAG6 (isoform 2) were cloned into pET30a in-frame with the His and S tags, and the protein expressed in *E. coli* as previously described (see Ref [25] in main text). Bacteria were lysed by sonication, the soluble fraction incubated with HisPur Cobalt resin (ThermoScientific) and, after extensive washing, the bound protein was eluted with buffer supplemented with the indicated concentrations of imidazole. Each fraction was analysed by SDS-PAGE and the gel stained with Coomassie Brilliant Blue. Full-length His-S-BAG6_1–321_ (BAG6 1–321aa) and its degradation products (BAG6_1–321_ degrad.) are indicated.
(TIF)

Figure S5 **Ubiquitin-like domains of UBL4A and BAG6 compete with His-S-BAG6_1–321_ for SGTA binding.** The same amount of immobilized SGTA was incubated with 2 µM BAG6_1–321_ alone (lane 1) or in the presence of increasing concentrations of recombinant UBLs derived from UBL4A (lanes 2 to 4) or BAG6 (lanes 5 to 7). The amount of BAG6_1–321_ recovered in each case was estimated by quantitative immunoblotting and expressed as a percentage of the recovery obtained in the absence of any competing UBL (lane 1). The estimated molar ratio of BAG6_1–321_ to recombinant UBL for each reaction is indicated. This is an independent repeat of the experiment presented in Figure 5F of the main text (see also Materials and Methods).
(TIF)
