# Supplementary figures and images for: The Association of BAG6 with SGTA and Tail-Anchored Proteins
Source: PLoS One. 2013 Mar 22;8(3):e59590. doi: 10.1371/journal.pone.0059590 (PMC3606182; doi:10.1371/journal.pone.0059590)

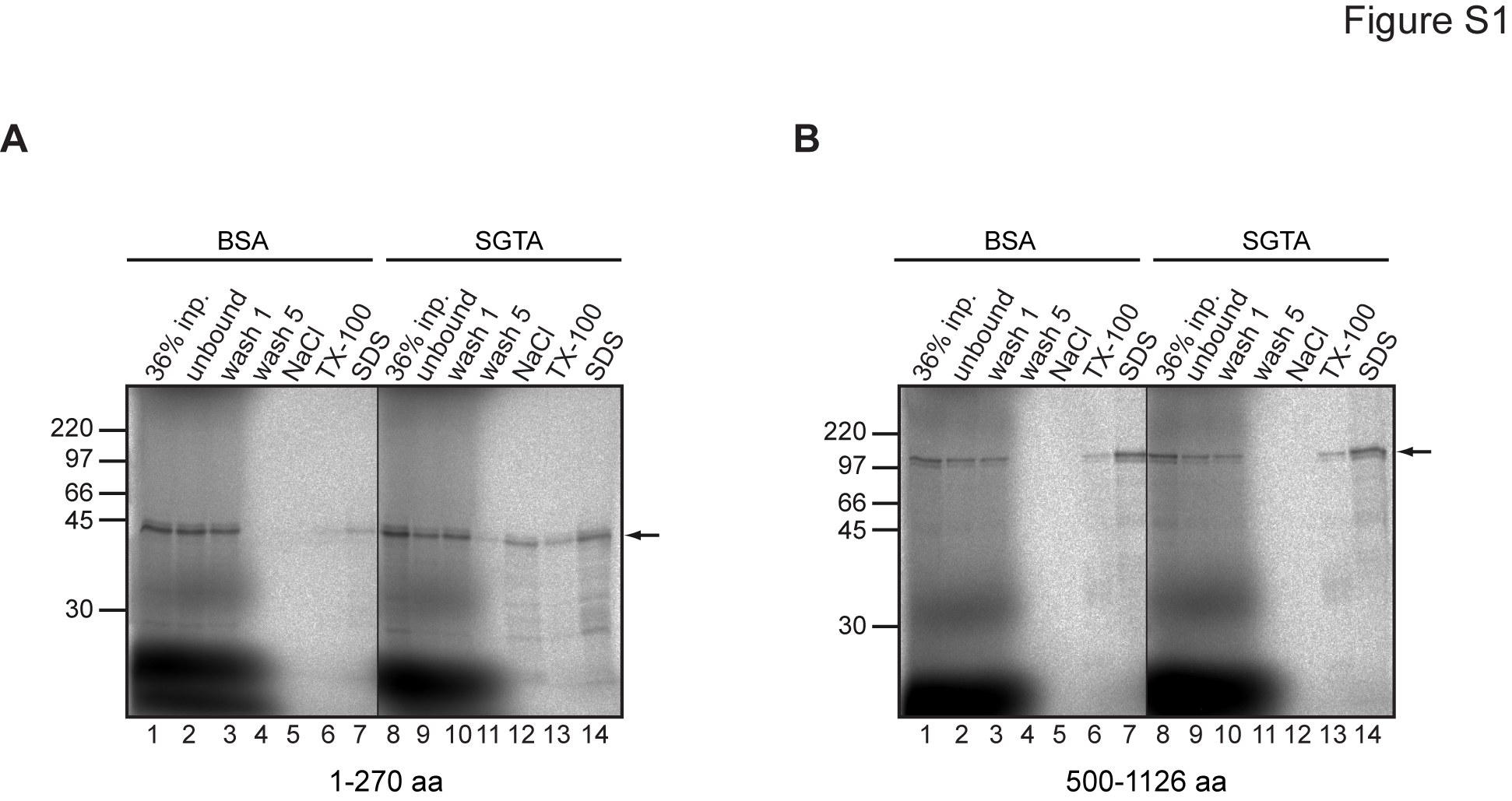

Supplement: Figure S1 — BAG6 associates with SGTA in a salt-sensitive manner. BAG6 fragments encompassing residues 1 to 270 (A) or 500 to 1126 (B) were synthesised in vitro using a wheat-germ extract and incubated with immobilized BSA or SGTA as shown (see Materials and Methods). Unbound material was collected, beads washed five times with low-salt buffer, followed by elution with buffer containing 1M NaCl, 0.5% (v/v) Triton X-100 and finally with SDS-PAGE sample buffer. A fraction of input, equivalent to 36% of the material used for the binding reaction, the unbound material, low salt washes 1 and 5 and the material eluted with NaCl, Triton X-100 and SDS-PAGE sample buffer were resolved by SDS-PAGE and the products visualized by phosphorimaging. An arrow indicates the location of the relevant translation product in each of the reactions. The same exposure of a single gel is shown with irrelevant lanes removed for clarity. As can be seen, bound material is eluted from immobilized SGTA with NaCl when the BAG6 fragment contains an intact N-terminal region (panel A, cf. lanes 5 and 12), but not when the N-terminal regions is absent (panel B, cf. lanes 5 and 12). (TIF) [file pone.0059590.s001.tif]

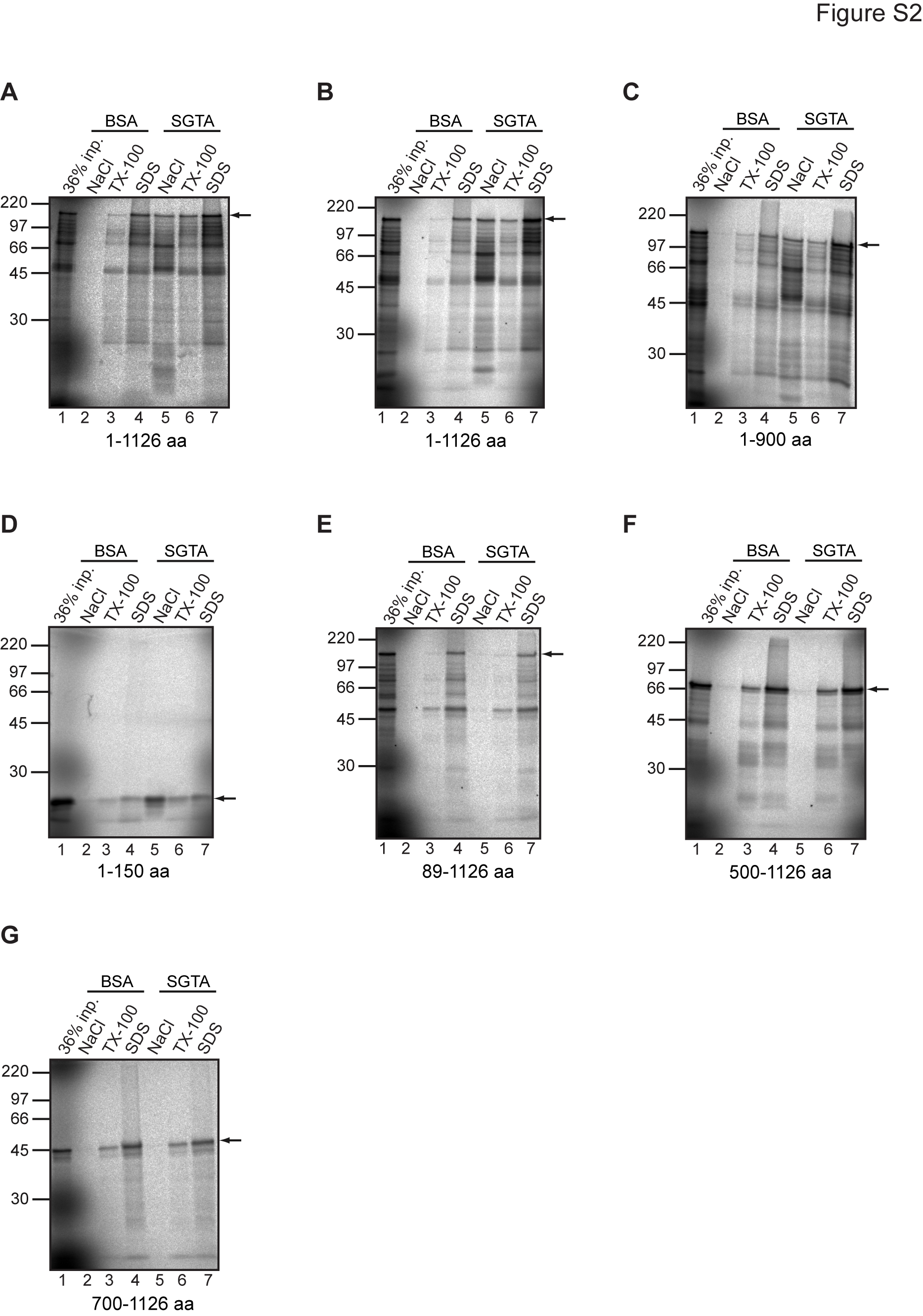

Supplement: Figure S2 — BAG6 interaction with SGTA is highly reproducible. Additional examples of the binding of full length BAG6, and BAG6 fragments, to immobilized BSA and SGTA are shown. These represent independent repeats of the qualitative pull down experiments presented in Figure 1 of the main text, and illustrate the highly reproducible nature of the salt sensitive interaction between fragments of BAG6 with an intact N-terminal UBL and SGTA. (TIF) [file pone.0059590.s002.tif]

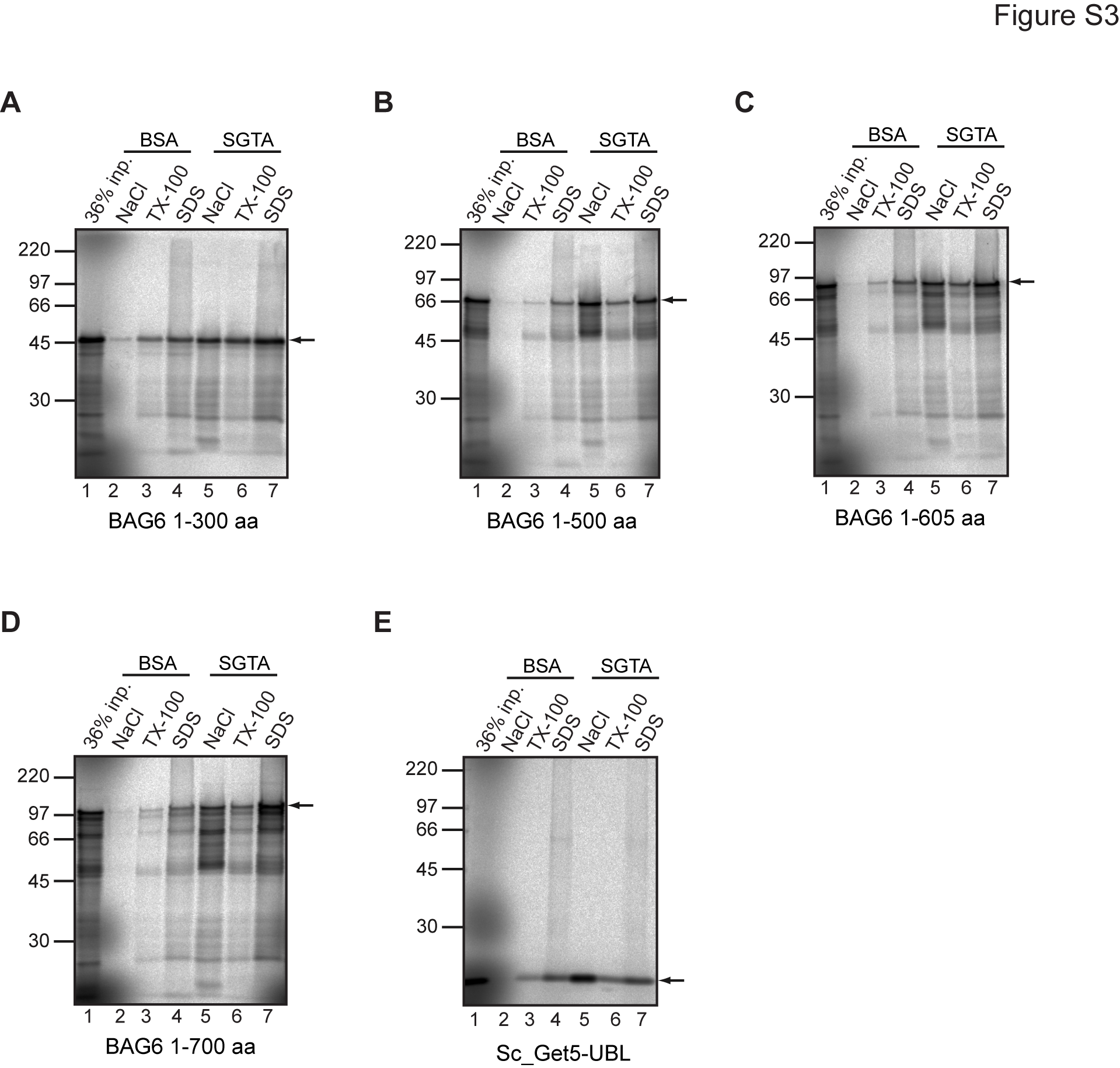

Supplement: Figure S3 — Analysis of SGTA interactions using additional BAG6 fragments and the UBL domain from S. cerevisiae GET5. Additional N-terminal fragments of BAG6 (A–D) that are not presented in the main text, and the UBL domain from S. cerevisiae GET5, Sc_Get5-UBL, (E; cf. main text, Fig. 3C) were translated in vitro using wheat-germ extract and their binding to immobilized BSA and SGTA analyzed as described for Figure 1 of the main text (see also Materials and Methods). We consistently observe that N-terminal fragments of BAG6 that contain up to 700 residues show an abnormal migration on SDS-PAGE, a behavior that most likely reflects the comparatively high proportion of proline residues located in this region of the protein. (TIF) [file pone.0059590.s003.tif]

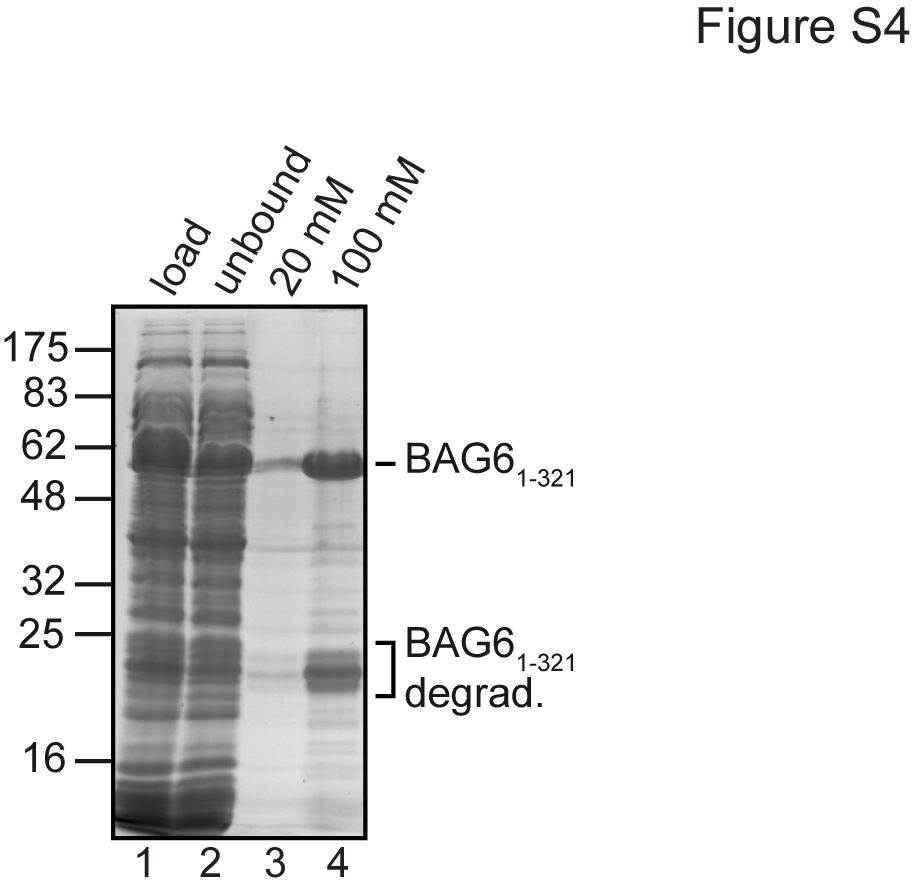

Supplement: Figure S4 — Purification of His-S-BAG61–321. N-terminal 1–321 residues of BAG6 (isoform 2) were cloned into pET30a in-frame with the His and S tags, and the protein expressed in E. coli as previously described (see Ref [25] in main text). Bacteria were lysed by sonication, the soluble fraction incubated with HisPur Cobalt resin (ThermoScientific) and, after extensive washing, the bound protein was eluted with buffer supplemented with the indicated concentrations of imidazole. Each fraction was analysed by SDS-PAGE and the gel stained with Coomassie Brilliant Blue. Full-length His-S-BAG61–321 (BAG6 1–321aa) and its degradation products (BAG61–321 degrad.) are indicated. (TIF) [file pone.0059590.s004.tif]

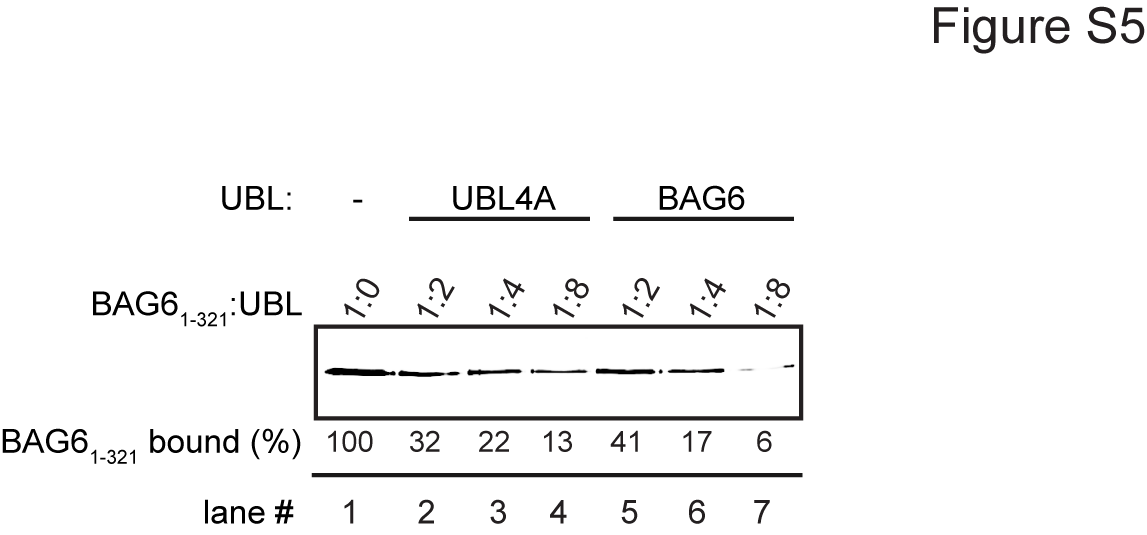

Supplement: Figure S5 — Ubiquitin-like domains of UBL4A and BAG6 compete with His-S-BAG61–321 for SGTA binding. The same amount of immobilized SGTA was incubated with 2 µM BAG61–321 alone (lane 1) or in the presence of increasing concentrations of recombinant UBLs derived from UBL4A (lanes 2 to 4) or BAG6 (lanes 5 to 7). The amount of BAG61–321 recovered in each case was estimated by quantitative immunoblotting and expressed as a percentage of the recovery obtained in the absence of any competing UBL (lane 1). The estimated molar ratio of BAG61–321 to recombinant UBL for each reaction is indicated. This is an independent repeat of the experiment presented in Figure 5F of the main text (see also Materials and Methods). (TIF) [file pone.0059590.s005.tif]
